# Generative AI for pre-consultation mental health triage in disorders of gut-brain interaction

**DOI:** 10.3389/fpsyt.2026.1854670

**Published:** 2026-06-16

**Authors:** Dakai Zeng, He Zeng, Jie Song, Kai Zhu

**Affiliations:** 1 Third Affiliated Hospital of Wenzhou Medical University, Wenzhou, China; 2 Ruian Fifth People’s Hospital, Wenzhou, China

**Keywords:** disorders of gut-brain interaction, generative AI, mental health, multidomain assessment, pre-consultation triage, psychogastroenterology

## Abstract

Disorders of gut-brain interaction (DGBI) are common, disabling, and frequently accompanied by anxiety, depressive symptoms, sleep disturbance, symptom-related fear, and repeated health care use. In routine gastroenterology practice, these problems are often recognized late, after fragmented histories, multiple visits, and avoidable investigations. Recent work on generative artificial intelligence (GenAI) and conversational systems suggests a narrower and more practical clinical use case than autonomous diagnosis: supervised pre-consultation triage. We propose that DGBI is a suitable setting for this approach because triage depends on integrating symptom narratives, prior investigations, alarm features, and psychosocial context rather than on a single test result. A GenAI-enabled intake tool could summarize patient-entered histories, incorporate brief distress screening and symptom diaries, flag possible medical or psychiatric escalation, and help route patients toward standard gastroenterology review, integrated psychogastroenterology, dietetic input, or urgent assessment. Its value would lie in making the first consultation more efficient and more clinically informed, not in replacing specialist judgment. For such systems to be acceptable, five conditions are essential: a narrowly defined triage task, multidomain but proportionate data collection, explicit rules for medical and psychiatric escalation, clinician review before action, and prospective evaluation across workflow, safety, equity, and patient acceptability. DGBI offers a realistic opportunity to develop GenAI tools that are useful precisely because they are constrained, auditable, and embedded in multidisciplinary care.

## 1 Introduction

Disorders of gut-brain interaction (DGBI) are among the most common conditions in gastroenterology, yet care remains constrained by fragmented pathways, short consultations, and uneven access to integrated psychological expertise. Recent overviews emphasize that illness burden is not captured by symptom counts alone, because abdominal pain, altered bowel habits, bloating, nausea, and related gastrointestinal symptoms often interact with stress, sleep, diet, illness beliefs, and prior healthcare experiences (1, 2). For many patients, the burden extends to work, eating behavior, social participation, and confidence in bodily control. Keefer et al. (3) and Knowles et al. (4) further note that anxiety, depressive symptoms, trauma-related processes, somatic hypervigilance, and symptom-driven avoidance can shape illness severity, help-seeking, and treatment response.

The first gastroenterology consultation is therefore expected to accomplish a great deal. Clinicians must assess alarm features, judge whether a DGBI diagnosis is likely, identify overlapping conditions, review prior investigations and treatments, explore psychosocial contributors, explain the gut-brain model, and formulate a management plan. In practice, this often begins with a sparse referral letter and a brief verbal history. When symptom, behavioral, and psychosocial information is incomplete at first review, testing may be inefficient and psychological needs may be recognized late, a front-end problem underscored by Guadagnoli et al. (5) and Staudacher et al. (6).

Against this background, generative artificial intelligence (GenAI) has attracted growing attention across medicine. In DGBI, however, the most defensible near-term role is not autonomous diagnosis, replacement of specialist judgment, or speculative inference of hidden psychopathology. Rather, it is a bounded assistive role in pre-consultation triage: organizing symptom, psychosocial, and contextual information already provided by patients into a concise, reviewable summary for clinicians. That use case is more consistent with current work on narrow, supervised clinical AI than with expansive claims about automation (7, 8). Heinz et al. (9) and Malgaroli et al. (10) likewise caution that mental health-related AI tools require clear limits, clinician oversight, and careful attention to context.

**Figure 1:**
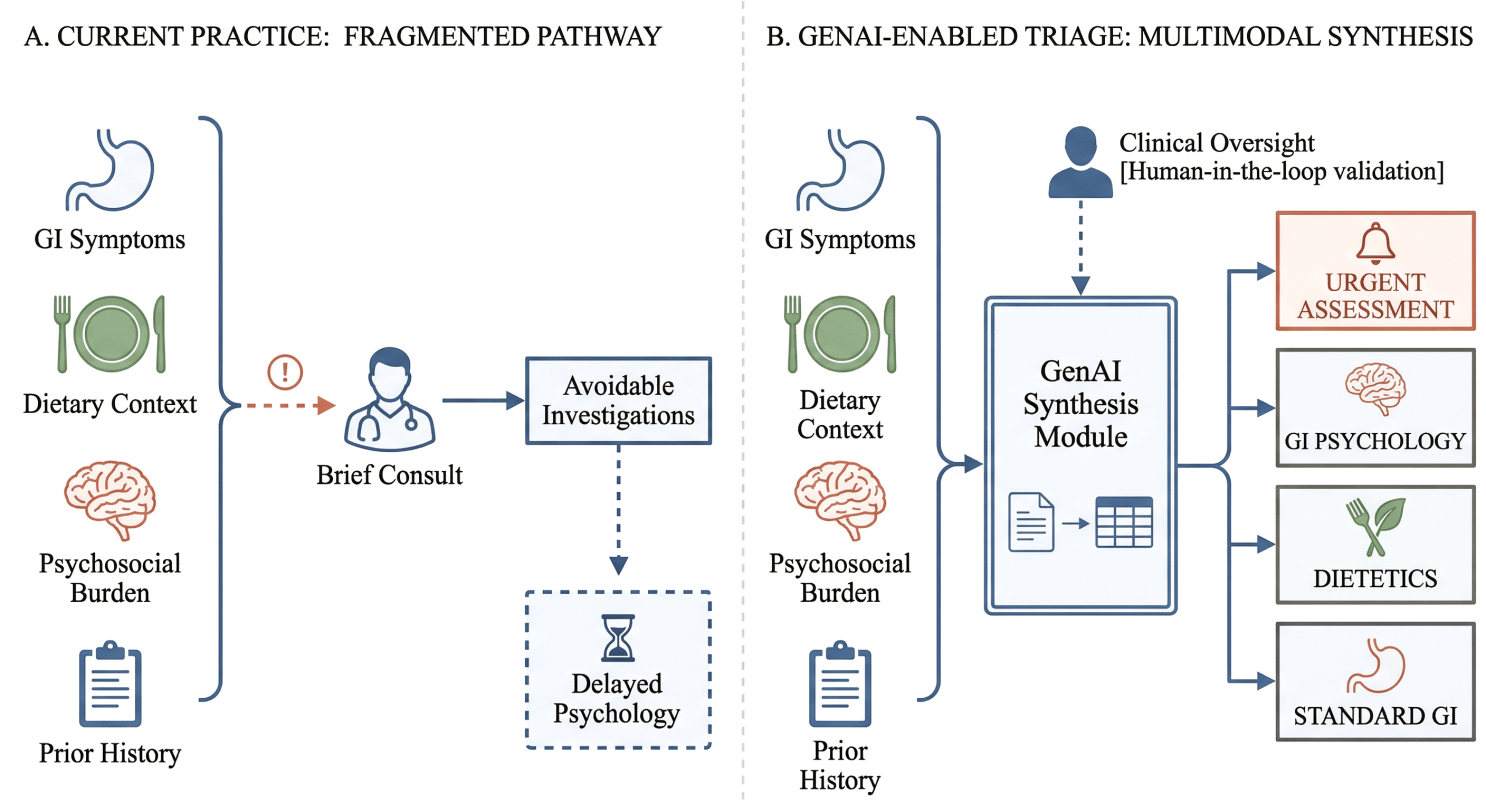
Comparison of the current fragmented clinical pathway **(A)** versus the proposed GenAI-enabled multidomain triage model **(B)** in DGBI care.

A pre-consultation model is attractive because it acts before the specialist encounter, when there is still an opportunity to route patients appropriately, gather missing information, and shape expectations. Yet the clinical threshold must remain high. Patients with DGBI may report severe distress even when urgent structural disease is unlikely, and mental health symptoms may be clinically relevant without being the principal driver of illness. Any triage system in this setting must therefore support nuance rather than reinforce simplistic binaries such as functional versus organic or medical versus psychological, tensions already recognized in DGBI care (1, 3). In this Perspective, we argue that DGBI is a suitable setting for GenAI-enabled pre-consultation triage, provided that the system is proportionate in scope, explicitly supervised by clinicians, and embedded in an implementation framework. In current practice, patient history is often fragmented, leading to delayed psychological recognition and avoidable investigations (see **Figure 1A**). The proposed GenAI-enabled triage model seeks to streamline this by providing clinical oversight and multidisciplinary routing at the point of entry (see **Figure 1B**).

## 2 Why DGBI is a suitable setting for pre-consultation triage

Several features of DGBI make it a strong candidate for carefully designed pre-consultation triage. First, these disorders are defined less by a single biomarker than by symptom pattern, chronicity, functional consequences, and contextual modifiers. Barbara et al. (1) and Zia et al. (2) make clear that DGBI assessment depends on how symptoms vary, what patients fear they signify, whether meals worsen them, how stress and sleep interact with symptoms, and what avoidance behaviors develop around eating, travel, or work. This pattern-rich, context-dependent history is central to DGBI diagnosis and management, yet it is often poorly captured in standard referral pathways.

Second, DGBI management benefits from early recognition of psychosocial processes that influence symptom burden and treatment engagement. These include not only general distress, but also bowel-specific anxiety, catastrophic symptom interpretation, food-related fear, trauma-related responses, and reduced trust in bodily control. Identifying such features does not mean psychologizing symptoms. Rather, it helps clinicians explain the disorder more effectively, assess whether gut-directed psychological therapies may be useful, and anticipate barriers to care. Keefer et al. (3) and Knowles et al. (4) argue that these processes are clinically meaningful because they shape symptom amplification, avoidance, and adherence even when they do not fully explain illness.

Third, DGBI services already operate under constrained time and stratified need. Some patients mainly require diagnostic confidence, explanation, and first-line management. Others present with severe symptom burden, repeated healthcare use, major functional impairment, prior negative investigations, or coexisting mental health concerns that justify earlier multidisciplinary input. A pre-consultation triage process can support this differentiation by identifying patients suitable for standard gastroenterology assessment, those who may benefit from earlier dietetic or psychological involvement, and those whose presentation requires more urgent escalation. This would formalize decisions that many services already make informally, often under time pressure (5, 6).

Finally, DGBI is a domain in which narrative matters. Patients often describe long diagnostic journeys, prior dismissal, and uncertainty about whether their symptoms are believed. How information is elicited and summarized can therefore affect trust. If the first consultation begins with evidence that symptom patterns, fears, and daily consequences have already been heard, the encounter may be more productive. GenAI may be useful here not as a source of new knowledge, but as a tool for synthesizing patient-entered information into a clinically usable summary while preserving the patient’s voice, a role more aligned with assistive than autonomous clinical AI (7, 8). Because the task can be narrowly defined around alarm features, symptom pattern, psychosocial complexity, and routing urgency, it is also more governable than open-ended diagnostic AI, as Bedi et al. (11) and the World Health Organization (12) would support.

The core features that make DGBI suitable for pre-consultation triage—narrative-rich assessment, psychosocial complexity, stratified service need, and the importance of early patient validation—are not unique to DGBI. Similar arguments could be made for chronic pain, fibromyalgia, and functional neurological disorder, all of which share symptom patterns shaped by behavioral, cognitive, and social processes and all of which face recognition delays and fragmented care. We acknowledge these analogues and note that the proposed triage framework may generalize to these conditions. However, DGBI offers a pragmatic starting point for three reasons. First, psychogastroenterology already exists as a defined integrative subspecialty with established brain-gut behavior therapy frameworks (3), providing a clinical infrastructure into which a triage tool could be embedded without building *de novo* services. Second, the Rome IV diagnostic taxonomy offers a consensus nosology that anchors clinical reasoning, reducing the risk that algorithmic triage decisions will be made without a shared diagnostic framework. Third, DGBI services are increasingly organized around multidisciplinary models that include gastroenterology, dietetics, and psychological care, providing natural routing endpoints for a triage system. These structural features, rather than any inherent uniqueness of gut-brain pathophysiology, make DGBI a tractable proving ground for the approach described here, with potential lessons for analogous conditions once the model is established.

## 3 A pragmatic framework for GenAI-enabled triage

**Figure 2:**
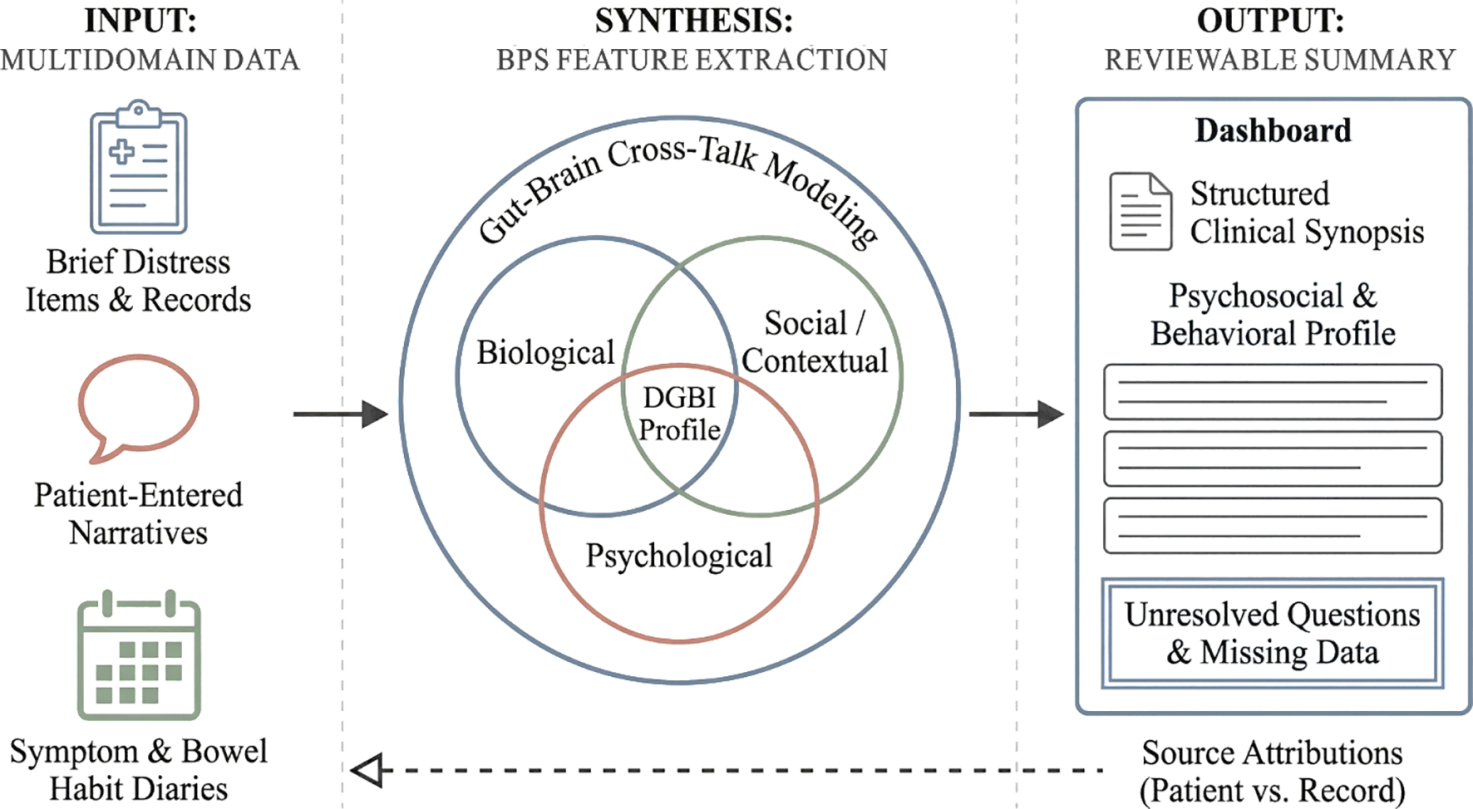
Conceptual framework for GenAI-enabled synthesis of multidomain patient data, illustrating the extraction of biopsychosocial (BPS) features to generate a reviewable clinical dashboard.

To move beyond generic symptom sorting, we propose a multidomain but proportionate approach to data collection (see **Figure 2**). This framework synthesizes patient narratives and diaries into a structured clinical synopsis that captures the gut-brain cross-talk central to DGBI. The operational stages, typical data inputs, and essential clinical safeguards for this model are summarized in **Table 1**.

**Table 1:** Proposed workflow and safeguards for GenAI-enabled pre-consultation mental health triage in DGBI.

Workflow stage	Clinical purpose	Typical inputs or outputs	Required safeguards
1. Patient intake	Capture the reason for referral, symptom chronology, alarm features, prior tests, and psychosocial burden before the visit	Patient-entered symptom narrative; brief distress items; bowel habit or symptom diary; uploaded records	Required questions for alarm features; clear statement that the tool does not provide diagnosis or emergency advice; immediate redirection to urgent care instructions when needed
2. Model-assisted synthesis	Transform unstructured information into a concise triage summary	Structured synopsis of GI symptoms, symptom impact, mental health burden, and unresolved questions	Use only clinically justified data sources; preserve provenance of model-generated inferences; avoid mandatory audio or video capture in routine care
3. Provisional routing	Suggest the most appropriate next step for review	Standard GI review; earlier clinician review; dietetic input; psychogastroenterology referral; urgent human contact	Rule-based medical and psychiatric escalation; no autonomous scheduling or referral without human confirmation
4. Clinician review	Confirm, revise, or override the model output before action	Triage summary reviewed by nurse or clinician alongside original patient text	Human-in-the-loop review before messages, booking changes, or referrals are sent; documentation of overrides
5. Follow-up and audit	Monitor whether the tool improves care and whether errors are systematic	Referral uptake; override rate; patient acceptability; no-show rate; urgent re-triage; subgroup performance	Prospective evaluation; bias monitoring; periodic model review; governance for updates, incident review, and data protection

### 3.1 What information should be collected

A useful triage framework should begin with the service rather than the model. The central question is not what GenAI can extract from unlimited data, but what minimum set of clinically justified information a gastroenterology-led service needs before first review. Excessive data collection would burden patients, generate noise, and create an illusion of precision. Too little information would reduce triage to generic symptom sorting. The most workable approach is therefore multidomain but proportionate, clinically interpretable, and aligned with decisions the service can actually make.

The core input should remain a structured pre-consultation questionnaire completed by the patient, ideally using plain language and branching logic to reduce unnecessary burden. Essential domains include symptom phenotype, duration, severity, bowel pattern, meal-related effects, nocturnal symptoms, weight change, gastrointestinal bleeding, vomiting, dysphagia, family history, prior investigations, current medications, and treatments already tried. These are familiar clinical elements, but triage depends on collecting them consistently and making alarm features explicit.

For DGBI, the intake should also go beyond symptom lists. It should ask about symptom-related avoidance, effects on eating, work or school disruption, sleep disturbance, fear of leaving home because of bowel urgency or pain, and interference with daily activities. Mental health assessment should likewise be targeted rather than exhaustive. The aim is not to conduct psychiatric diagnosis at intake, but to identify distress, comorbidity, and risk that are clinically relevant to gastroenterology care. Keefer et al. (3) and Knowles et al. (4) support this focused psychogastroenterology approach.

The proposed GenAI intake is intended to supplement, not replace, validated screening instruments already in use in DGBI and adjacent services—including the PHQ-9, GAD-7, Visceral Sensitivity Index, IBS-Symptom Severity Score, and the Rome IV diagnostic questionnaire. These instruments provide standardized severity benchmarks that are essential for clinical communication, longitudinal tracking, and comparison across services. GenAI synthesis adds contextual richness that structured instruments alone cannot capture: a PHQ-9 score of 14 conveys severity, but does not reveal whether low mood is driven by symptom unpredictability, social withdrawal from bowel urgency, or pre-existing depression. The natural design choice is integration rather than competition: structured instrument scores can be entered as part of the intake and serve as structured inputs to the GenAI synthesis step, while free-text elaboration captures the lived experience around those scores. Services transitioning to GenAI-supported intake need not abandon their existing screening workflows; the tool should be designed to ingest validated instrument data alongside narrative content, producing a synthesis that is richer than either modality alone.

Free-text fields remain important because patients often express concerns that no checkbox captures, such as fear of cancer despite prior normal tests, embarrassment about incontinence, panic in public settings, or frustration after repeated dismissal. GenAI may help summarize such narratives, but the original text should remain visible to the clinician, because Li et al. (8) and Malgaroli et al. (10) both caution against overinterpreting text without preserving context. The purpose of summarization is not to overwrite the patient’s account; it is to extract concerns, illness beliefs, functional effects, and barriers to engagement in a way that is easier to review.

A multidomain approach may also incorporate referral letters, previous clinic notes, medication history, and patient-reported outcome measures where available. In principle, wearables, food logs, or symptom diaries could add context, but they should not be core requirements for routine triage. Emerging work on digital symptom monitoring in gastrointestinal disorders suggests that patient-reported outcome data collected between visits can provide clinically informative context (6), yet Warraich et al. (13) also make clear that uneven access and implementation burden can widen inequities. A proportionate model should therefore prioritize information that is routinely obtainable, understandable to clinicians, and actionable at first review. The intake must also include a clear safety layer: reports of self-harm, suicidal ideation, severe depression, restrictive eating with nutritional risk, inability to maintain hydration, safeguarding concerns, or gastrointestinal alarm symptoms should trigger predefined escalation pathways with human review rather than remain inside ordinary triage logic (11, 12).

At minimum, a pre-agreed escalation protocol should specify: (a) the named clinical role responsible for review (e.g., duty gastroenterologist, clinical nurse specialist, or on-call liaison psychiatry); (b) a maximum review window (e.g., within 4 hours during working hours, or a defined out-of-hours pathway); (c) the clinical channel through which escalation occurs (e.g., direct notification to the electronic health record task list with audit trail, or direct phone contact for imminent risk); and (d) a documented fallback procedure when the primary reviewer is unavailable, including escalation to the next designated role. These parameters should be agreed before deployment and audited periodically. Services that cannot commit to these minimum elements should not collect high-risk mental health information through automated intake, as unactioned risk disclosure may paradoxically worsen patient safety.

### 3.2 What the output should contain

The output of a GenAI-enabled triage system should be modest, structured, and clinically legible. It should not provide a definitive diagnosis, a psychiatric formulation, or a recommendation that bypasses clinician judgment. A useful output might include a concise summary of gastrointestinal symptoms and functional impact, a section for alarm features or missing data, a psychosocial and behavioral profile relevant to DGBI care, a suggested triage category with transparent rationale, and a short list of questions or actions for the reviewing clinician.

Transparency is essential. The summary should indicate which statements derive from patient report, which from referral documents, and where uncertainty remains. If the system highlights meal-related symptoms, panic linked to bowel urgency, or prominent illness concern, the source material should be reviewable. Chang et al. (7) emphasize the risk of fluent but unsupported clinical summaries, and the CHART Collaborative (14) similarly underscores the value of traceable outputs. This is especially important when psychological factors are summarized. A label such as high anxiety is too imprecise unless anchored to specific patient-reported experiences.

We acknowledge a practical tension: the paper argues both that the original patient text should remain visible to clinicians and that the AI summary should make information easier to review. Under time pressure, clinicians may default to the summary, potentially missing nuance that the tool omitted or misrepresented—particularly in psychosocial content where tone, phrasing, and narrative structure carry clinical meaning that a summary may flatten. This tension should be addressed through interface design rather than left to clinician discipline. We propose three design responses. First, a mandatory viewing step: for psychosocial content that the system flags as clinically salient (e.g., distress indicators, trauma-related disclosures, eating-related concerns), the original patient text should be displayed before the summary, and the clinician should be required to acknowledge it before proceeding to routing. Second, a mismatch flag: when the synthesis step has low confidence in a psychosocial attribution—for example, when patient language is ambiguous or contradictory—a visible flag should alert the reviewing clinician to consult the original text directly. Third, a required override step: routing decisions that involve mental health-related triage categories should require an explicit clinician confirmation that the original text has been reviewed, not merely that the summary appears plausible. These design features make the tension visible and correctable within the workflow rather than relying on clinician vigilance alone.

The output should also be designed to avoid common errors. One is conflating likely DGBI with exclusion of organic disease. Another is reducing mental health information to a single distress score that obscures mechanism and clinical meaning. A third is generating recommendations that the service cannot realistically implement. If a clinic has no embedded psychologist, the triage output should not routinely prescribe unavailable interventions. It may instead indicate that psychogastroenterology-informed explanation, stepped psychological referral, or coordination with primary care mental health services should be considered, which is more consistent with realistic service design (5). It is also useful to separate clinical summary from operational routing so that multidisciplinary teams receive actionable information rather than a generic label of high complexity.

### 3.3 Workflow integration and human oversight

The value of pre-consultation triage depends less on model performance in isolation than on how the output enters everyday practice. In practical terms, triage should be reviewed by a named clinician or appropriately trained team member before appointment allocation or major pathway decisions are made. The human reviewer remains responsible for deciding whether the summary is accurate enough to inform routing and whether escalation is needed. That oversight is part of what keeps the use case bounded and clinically accountable, a point consistent with governance recommendations from Bedi et al. (11) and the World Health Organization (12).

Workflow design should begin with the patient journey. After referral, patients could receive a digital intake package explaining why information is being collected, what role the tool plays, how privacy is handled, and what to do if urgent concerns arise. Completion should not be mandatory when barriers exist. Paper, telephone, or assisted completion routes are needed for patients with limited digital access, language barriers, low health literacy, or symptom severity that makes extensive questionnaires difficult. Vasey et al. (15) and Warraich et al. (13) both support this attention to implementation context. Once intake is completed, the system can generate a draft summary for review, but no patient-facing conclusions should be issued without clinician approval.

Data governance for this application requires particular care given the sensitivity of collected information. Three principles should guide design. First, informed consent: patients should receive clear, plain-language explanation of what data are collected, how AI-generated summaries are produced, where data are stored, who can access them, and under what circumstances information may be shared with other providers. Consent should cover the use of de-identified data for service evaluation and model refinement, with opt-out mechanisms that do not affect clinical care. Second, data minimization: the intake should collect only information that is clinically justified for pre-consultation triage, and AI-generated summaries should be treated as clinical decision-support outputs subject to the same retention, correction, and access policies as other elements of the health record. The regulatory status of AI-generated clinical summaries remains in flux across jurisdictions, but the principle should be that summaries are part of the clinical record and subject to the same privacy and security obligations as any other clinical documentation. Under frameworks such as HIPAA and GDPR, this implies encryption at rest and in transit, role-based access control, audit logging, and defined data retention and deletion schedules. Third, storage and deletion: services must define retention periods for intake data and AI-generated summaries, specify deletion procedures that include backup systems and model training corpora, and ensure that patients can request correction or removal of misattributed content in accordance with applicable data subject rights. These provisions should be documented in a data protection impact assessment before any system collecting mental health disclosures is deployed, consistent with the staged evaluation approach described in Section 5.

Within the service, triage outputs should support specific decisions: whether an appointment needs expedited review, whether additional records or tests should be obtained beforehand, whether the first visit should be longer, and whether parallel input from dietetics or psychology may be useful. A patient with severe bowel-specific anxiety, repeated urgent care use, and avoidance of leaving home may benefit from a first consultation that emphasizes validation and explanation even if no urgent organic concern is identified. A patient with progressive dysphagia, weight loss, and vomiting should bypass routine DGBI-oriented triage. Systems should also make clinician correction straightforward. If users must routinely rewrite summaries or work around the tool, adoption will fail; as the CHART Collaborative (14) and Vasey et al. (15) imply, iterative refinement with feedback is therefore essential.

Illustrative case. Consider a 34-year-old woman referred to gastroenterology with a 2-year history of abdominal pain, bloating, and alternating bowel habit. She has undergone colonoscopy and CT imaging, both unremarkable. She reports that meals worsen her pain, that she has stopped eating at restaurants and social events, and that she fears her symptoms indicate a missed serious disease. She describes poor sleep on nights before work, panic-like sensations when bowel urgency strikes in public, and frustration that previous clinicians told her that ‘everything is normal.’ Her PHQ-9 score is 11 and GAD-7 is 13. She does not report suicidal ideation, self-harm, or alarm features. At Stage 1 (intake), the system collects her symptom narrative, diary entries, PHQ-9 and GAD-7 scores, and free-text elaboration about social withdrawal and symptom-related fear. At Stage 2 (synthesis), the model produces a structured synopsis: Rome IV-compatible IBS-M pattern, moderate generalized anxiety with prominent bowel-specific anxiety features, significant functional impairment (social avoidance, work-related anticipatory anxiety), no alarm features, no suicidal ideation. Each psychosocial attribution is anchored to her specific statements. At Stage 3 (routing), the system suggests a provisional triage category of ‘integrated psychogastroenterology review’ with rationale citing the combination of functional impairment, bowel-specific anxiety, and moderate distress scores, alongside the recommendation that initial consultation include gut-brain explanation and assessment for brain-gut behavior therapy suitability. At Stage 4 (clinician review), the reviewing clinician confirms the summary accuracy after reviewing the original text, notes that dietetic input may also be useful, and schedules a longer first appointment. At Stage 5 (audit), the case is logged for service evaluation, with override documentation and patient acceptability assessed post-consultation. This illustrative case demonstrates how the framework differentiates structured information from narrative context, anchors psychosocial inference to patient report, preserves clinician authority at the routing decision, and maintains auditability.

## 4 Safety, equity, and evaluation

Safety in GenAI-enabled triage extends beyond fluent summarization. A system may produce polished output yet still create risk by normalizing false reassurance, reproducing stigmatizing assumptions, or delaying urgent review. In DGBI, where patients are especially vulnerable to being told that symptoms are just stress, the ethical threshold is high. Psychological information should enrich care, not displace biomedical vigilance. This requires clear separation of alarm-feature handling from psychosocial complexity profiling, mandatory review of flagged cases, and audit of false reassurance events. Chang et al. (7)Bedi et al. (11), and the World Health Organization (12) all point toward this broader view of safety.

Mental health safety requires equal attention. Pre-consultation intake may reveal suicidal ideation, severe panic, trauma-related symptoms, self-neglect, or restrictive eating that was not evident in the referral. A service that asks these questions assumes responsibility for acting on the answers. It is not acceptable to collect high-risk information without clear escalation pathways, defined review time frames, and named clinical responsibility. Heinz et al. (9) highlight the high-stakes nature of mental health-related AI deployment, and the World Health Organization (12) similarly supports conservative escalation thresholds linked to existing mental health or emergency systems. As noted in Section 3.1, this responsibility requires operational specificity: named reviewers, defined time frames, explicit clinical channels, and documented fallback procedures must be agreed before any system that collects such information is deployed.

In DGBI triage, the most clinically specific hallucination risk arises during psychosocial summary generation. A fluent but inaccurate GenAI summary attributing anxiety, catastrophizing, or trauma-related features to a patient who did not report them, or misrepresenting the severity of suicidal ideation, could cause clinical harm through incorrect routing or false reassurance. Several design constraints can reduce this risk. First, the synthesis step should be constrained to extractive or template-bound summarization, in which each psychosocial claim is explicitly anchored to a patient-reported statement, rather than free-form narrative generation. Second, all model-generated psychosocial labels should require disambiguation: if a patient describes temporary worry about a procedure, the system must not conflate this with generalized anxiety unless corroborated by additional responses. Third, the summary interface should flag model-generated inferences explicitly and present source text alongside each psychosocial attribution, enabling clinicians to verify accuracy at a glance. Fourth, escalation-sensitive content—suicidal ideation, self-harm, severe nutritional risk—should bypass the generative synthesis step entirely and follow deterministic, rule-based routing, as LLM-generated paraphrases of safety-critical disclosures introduce unacceptable risk. These safeguards make hallucination visible and correctable within the clinician-review workflow, consistent with the broader safety framework already described.

We reiterate that the data governance measures outlined in Section 3.3—informed consent, data minimization, and defined retention and deletion obligations—are integral to the safety framework. Collecting sensitive psychosocial information without corresponding governance infrastructure is itself a safety risk.

Equity concerns are substantial. Patients who are older, socioeconomically disadvantaged, non-native speakers, neurodivergent, visually impaired, or less comfortable with digital tools may provide shorter or less polished responses. If triage quality depends on narrative fluency or culturally normative expressions of distress, underassessment may worsen in groups already at risk. Missingness, brevity, or linguistic difference should therefore not be read as low complexity. Vasey et al. (15) warn against deployment that ignores subgroup performance, and Warraich et al. (13) stress that implementation choices can deepen inequity if support routes are not built in.

Bias may also arise from training data and local clinical habits. If prior records disproportionately frame women, younger patients, or those with anxiety diagnoses as primarily functional, automated summarization may inherit those patterns. The same risk applies to patients with chronic pain syndromes or high healthcare use. Responsible implementation therefore requires periodic fairness audits that assess not only model outputs but downstream decisions such as urgency assignment, referral to psychology, investigation patterns, and patient-reported experience. That emphasis on downstream impact follows naturally from the reporting and governance priorities set out by Vasey et al. (15) and the CHART Collaborative (14).

Evaluation should proceed in phases. Initial work should focus on feasibility, completion rates, acceptability, and agreement between generated summaries and clinician review. Subsequent studies can assess operational outcomes such as preparation time for first consultations, completeness of key history domains, identification of urgent concerns, and appropriateness of routing. Only after these process measures are established should broader questions be asked about patient benefit. The CHART Collaborative (14) supports this staged approach, and Warraich et al. (13) make clear that qualitative evaluation also matters because patients, clinicians, and administrative teams may each experience the tool differently.

To make the phased evaluation framework actionable, candidate outcome measures and illustrative progression thresholds should be specified at each stage. Phase 1 (feasibility and safety): primary measures include intake completion rate (candidate threshold: ≥75% of referred patients completing intake without assistance, with defined subgroup monitoring), patient acceptability score on a brief post-intake survey (candidate threshold: mean score ≥4 on a 5-point Likert scale), inter-rater agreement between GenAI-generated summaries and independent clinician review of the same source material (candidate threshold: Cohen’s κ ≥0.60 for triage category assignment), and adverse event rate (any documented instance of false reassurance, missed alarm feature, or inappropriate psychosocial attribution requiring correction; candidate threshold: zero events prompting clinical harm). Phase 2 (operational outcomes): measures include clinician-rated preparation time saved per first consultation, completeness of key history domains compared with standard referral, appropriate identification of cases requiring expedited review (sensitivity and specificity against clinician adjudication), and acceptability to clinical and administrative staff using a validated implementation measure such as the Acceptability of Intervention Measure. Phase 3 (patient benefit): measures include reduction in time from referral to appropriate multidisciplinary input, change in repeated low-yield investigations or visits, patient-reported experience measures (e.g., CollaboRATE or similar shared decision-making instrument), and equity audits that compare all preceding outcomes across age, language, digital literacy, ethnicity, and socioeconomic strata. These thresholds are illustrative and should be refined through multi-stakeholder consensus before protocol finalization.

## 5 Discussion

The promise of GenAI-enabled pre-consultation triage in DGBI is modest but potentially meaningful: improving the first specialist encounter by making symptom patterns, psychosocial context, and functional burden more visible before the visit. This is particularly relevant to a mental-health-focused special issue because DGBI sits at the intersection of gastroenterology and mental health, yet routine care often lacks structures that connect the two early enough. Keefer et al. (3) and Knowles et al. (4) show why that matters: clinical progress depends not only on diagnostic labeling but also on recognizing the behavioral and emotional processes that shape symptom experience and treatment engagement.

This approach should not be oversold. Many barriers in psychogastroenterology are structural rather than informational. Services may identify psychological need early and still lack referral capacity. Clinicians may receive better summaries but remain constrained by short appointments, fragmented records, and reimbursement models that favor investigation over explanation. Patients may disclose distress but resist mental health framing because of stigma or prior invalidation. GenAI cannot solve these problems. At best, it can reduce information loss and improve the timing and quality of clinical attention; whether that produces better outcomes depends on service design and institutional support (5, 6).

For that reason, implementation should be phased. The first phase should be conservative: structured intake, transparent summarization, and clinician-reviewed routing in selected DGBI pathways, without autonomous decision-making. The second phase can refine data elements, improve presentation, and test subgroup performance across language, literacy, and demographic strata. The third phase may examine whether integration with multidisciplinary services improves access, reduces repeated low-yield consultations, or supports earlier use of psychological and behavioral interventions. This staged logic is more consistent with responsible evaluation than with claims of rapid transformation (12, 14).

Clinician training deserves particular emphasis. A stronger pre-consultation summary is only useful if the receiving clinician knows how to use it. In DGBI, that means acknowledging distress without implying that symptoms are imagined, discussing gut-brain mechanisms without minimizing disease burden, and using mental health information to tailor care rather than exclude. Zia et al. (2) and Keefer et al. (3) both support this broader clinical stance. More broadly, DGBI care has long required clinicians to hold biological, psychological, and social information together without granting any one domain total explanatory authority. Pre-consultation triage should follow the same principle. If kept bounded, transparent, clinician-supervised, and equity-aware, GenAI may serve not as a substitute for psychogastroenterology, but as an operational aid that helps gastroenterology services respond to the mental health dimensions of DGBI earlier and more consistently.

## Data Availability

The original contributions presented in the study are included in the article/supplementary material. Further inquiries can be directed to the corresponding author.
